# Genetic characterization of carbapenem-resistant *Klebsiella pneumoniae* bloodstream isolates with reduced susceptibility to cefiderocol

**DOI:** 10.3389/fcimb.2026.1890499

**Published:** 2026-08-27

**Authors:** Min Wang, Xue Wu, Ning Yuan, Lin Li, Zhijun Zhang

**Affiliations:** 1 Department of Laboratory Medicine, The Affiliated Taian City Central Hospital of Qingdao University, Taian, China; 2 Shandong Provincial Key Medical and Health Laboratory of Anti-Drug Resistant Drug Research, The Affiliated Taian City Central Hospital of Qingdao University, Taian, China; 3 Department of Pain Management, The Affiliated Taian City Central Hospital of Qingdao University, Taian, China; 4 Binhai New Area Hospital of Traditional Chinese Medicine (TCM), Tianjin, China; 5 Pharmacy Intravenous Admixture Services, The Affiliated Taian City Central Hospital of Qingdao University, Taian, China

**Keywords:** bloodstream infection, carbapenem-resistant *Klebsiella pneumoniae*, cefiderocol, reduced susceptibility, ST11, virulence plasmid, whole-genome sequencing

## Abstract

**Objectives:**

To assess cefiderocol activity against carbapenem-resistant *Klebsiella pneumoniae* (CRKP) bloodstream isolates collected before local clinical introduction and to characterize the distribution of borderline MIC elevation across major genomic backgrounds.

**Methods:**

We retrospectively analyzed 389 episodes of *K. pneumoniae* bloodstream infection at a tertiary hospital in China during 2018-2024. All 83 carbapenem-resistant isolates underwent cefiderocol broth microdilution testing and whole-genome sequencing. For epidemiological analysis, reduced susceptibility was prespecified as an MIC of 4–16 mg/L and was not intended to replace clinical breakpoint interpretation.

**Results:**

CRKP accounted for 21.3% of *K. pneumoniae* bloodstream infections and remained associated with in-hospital mortality after adjustment for infection severity and source. By CLSI criteria, 83.1% of isolates were cefiderocol susceptible; the MIC_50_ and MIC_90_ were 4 and 8 mg/L, respectively, and 41.0% met the reduced-susceptibility definition. ST11 predominated, with KL47 and KL64 as the main capsular loci. Cefiderocol MICs were higher among KL47/KL64 and virulence-plasmid-associated isolates than among comparator backgrounds. In multivariable analysis, *bla*_NDM-1_, *bla*_SHV-12_, and the aerobactin locus remained associated with reduced susceptibility, although the findings require cautious interpretation because of limited sample size and possible effects of clonal background. No inactivating mutations were identified in *cirA*, *fepA*, or *fiu*.

**Conclusions:**

Borderline cefiderocol MIC elevation was present before local drug exposure and was more frequent in locally prevalent ST11-KL47/KL64 and virulence-plasmid-associated CRKP. These findings provide a bloodstream-specific pre-introduction baseline and support prospective surveillance of numerical MIC distributions and associated genomic backgrounds.

## 1 Introduction

*Klebsiella pneumoniae* is an important cause of bloodstream infection (BSI) and is frequently associated with septic shock, multiple organ failure, and substantial mortality (Li et al., 2023; Hassoun-Kheir et al., 2024). The global dissemination of carbapenem-resistant *K. pneumoniae* (CRKP) has further complicated clinical management and has been consistently associated with adverse outcomes, with reported mortality rates of 42%–81% in CRKP bloodstream infection (Wang M. et al., 2022; Wang Q. et al., 2024). As CRKP become increasingly prevalent, available treatments have become largely restricted to tigecycline, polymyxins, and newer β-lactam/β-lactamase inhibitor combinations such as ceftazidime-avibactam (CAZ-AVI) (Doi, 2019; Scudeller et al., 2021). Unfortunately, resistance to these last-line agents is now emerging rapidly within CRKP, and CAZ-AVI retains limited efficacy against class B β-lactamases, notably NDM enzymes (Shirley, 2018). Consequently, the development of novel effective antimicrobial alternatives is urgently required.

Cefiderocol (CFDC), a siderophore cephalosporin that enters bacterial cells through iron-uptake pathways, has emerged as a promising agent against carbapenem-resistant Gram-negative pathogens (Lewis et al., 2024). However, reduced susceptibility and resistance to CFDC have attracted increasing attention. High-level resistance is generally mediated by multiple coordinated mechanisms, including beta-lactamase overexpression, defects in siderophore receptors, porin abnormalities, and other permeability-related changes (Lan et al., 2022; Wang L. et al., 2024). By contrast, low-level CFDC resistance is more cryptic but may be clinically and epidemiologically important. Recent studies have shown that low-level CFDC resistance in CRKP is associated with NDM or KPC variants combined with high *bla*_SHV_ expression, and is frequently observed in ST11-KL64 strains carrying virulence plasmids (Hong et al., 2025). In addition, tandem amplification of *bla*_SHV-12_ may mediate heteroresistance and drive MIC increases under antibiotic pressure (Liu et al., 2024).

Importantly, CFDC susceptibility interpretation differs between CLSI and EUCAST. For Enterobacterales, CLSI classifies MICs of ≤4, 8, and ≥16 mg/L as susceptible, intermediate, and resistant, respectively, whereas EUCAST classifies MICs of ≤2 mg/L as susceptible and >2 mg/L as resistant. Thus, isolates with MICs of 4–8 mg/L are classified differently by CLSI and EUCAST, which may substantially affect reported non-susceptibility rates and clinical interpretation, particularly for NDM-producing *Klebsiella* bloodstream isolates (Clinical and Laboratory Standards Institute (CLSI), 2022; Isler et al., 2023; The European Committee on Antimicrobial Susceptibility Testing, 2026). Because CLSI standards are predominantly used in clinical practice in China, such isolates may be underestimated in routine testing and clinical decision-making. Under antimicrobial pressure, however, they may evolve further toward higher-level resistance, thereby increasing the risk of treatment failure and transmission (Liu et al., 2022). We therefore prespecified an MIC range of 4–16 mg/L as reduced susceptibility for epidemiological analysis of right-shifted MIC distributions, rather than as a clinical category intended to replace CLSI or EUCAST interpretation. Nevertheless, the baseline distribution of CFDC MICs among CRKP bloodstream isolates before routine local exposure, and the extent to which reduced susceptibility is enriched within locally dominant ST11-KL47/KL64 and virulence-plasmid-associated populations, remain insufficiently characterized.

CFDC has received regulatory approval from the U.S. Food and Drug Administration (FDA) and the European Medicines Agency (EMA), and was approved by the China National Medical Products Administration (NMPA) in January 2026. The present study therefore focused on all CRKP bloodstream isolates collected at a tertiary hospital during 2018–2024, before local clinical introduction of cefiderocol. By combining reference MIC testing, whole-genome sequencing, and patient-level data, we aimed to establish a pre-introduction baseline, characterize the distribution of reduced susceptibility across ST11-KL47/KL64 and virulence-plasmid-associated backgrounds, and assess whether previously reported genomic determinants remained associated after multivariable regression. This design was intended to provide an incremental, bloodstream-specific epidemiological contribution.

## 2 Materials and methods

### 2.1 Study design and definitions

This single-center retrospective observational study was conducted at Taian Central Hospital, a 2000-bed tertiary medical center in Shandong Province, China. All patients diagnosed with *K. pneumoniae* BSI between January 2018 and December 2024 were screened for inclusion. Patients were excluded if medical records were incomplete or if polymicrobial bloodstream infection was present. For patients with multiple positive blood cultures, only the first *K. pneumoniae* isolate was retained. Isolates were identified during routine clinical microbiological testing, stored at −80 °C in LB broth supplemented with 20% glycerol, and recovered on blood agar before further testing. Among the 389 non-duplicate bloodstream isolates included, 83 met the predefined criteria for carbapenem resistance and underwent cefiderocol susceptibility testing and whole-genome sequencing. The study was approved by the Ethics Committee of Taian Central Hospital.

Demographic and clinical data were extracted from the hospital electronic medical record system. The primary outcomes of interest were 30-day and in-hospital all-cause mortality. *K. pneumoniae* BSI was defined as at least one positive blood culture for *K. pneumoniae* accompanied by compatible clinical manifestations of BSI (Timsit et al., 2020). The date of collection of the first positive blood culture was defined as the onset date. Antibiotic exposure was defined as continuous antibiotic use for more than 48 h within 30 days before BSI onset or before discharge. Episodes were classified as catheter-related when a catheter had been in place for >48 h and no other infectious focus was identified. Episodes without a definite source were classified as unknown source. Hospital-acquired infection (HAI) was defined as a positive blood culture obtained >48 h after admission. If the infectious focus involved an indwelling device that had been in place for >48 h, the episode was classified as device-related infection.

### 2.2 Microbiological methods and antimicrobial susceptibility testing

Blood cultures were processed using the BD BACTEC™ FX400 system (Becton, Dickinson and Company, Franklin Lakes, NJ, USA). Species identification was performed using the Autof MS1000 MALDI-TOF mass spectrometry system (Autobio Diagnostics Co., Ltd., Zhengzhou, China), and routine antimicrobial susceptibility testing (AST) was performed using the VITEK-2 system (bioMérieux, France). The antimicrobial panel included cefazolin (KZ), cefuroxime (CXM), ceftazidime (CAZ), ceftriaxone (CRO), cefepime (FEP), ampicillin/sulbactam (SAM), cefoperazone/sulbactam (SCF), piperacillin/tazobactam (TZP), imipenem (IPM), meropenem (MEM), ertapenem (ETP), tobramycin (TOB), amikacin (AMK), gentamicin (GEN), ciprofloxacin (CIP), levofloxacin (LEV), trimethoprim/sulfamethoxazole (SXT), and aztreonam (ATM). MICs of colistin (Col), tigecycline (TGC), and ceftazidime/avibactam (CZA) were determined by broth microdilution. The MIC of CFDC was determined by broth microdilution in iron-depleted cation-adjusted Mueller–Hinton broth (ID-CAMHB) as described in CLSI M100, 32st ed (Clinical and Laboratory Standards Institute (CLSI), 2022). Cefiderocol powder was purchased from MedChemExpress (Monmouth Junction, NJ, USA; catalog no. HY-17628). ID-CAMHB was prepared from cation-adjusted Mueller–Hinton II broth (Shandong Tuopu Bio-engineering Co., Ltd., Zhaoyuan, Shandong, China; catalog no. M8114B) using Chelex^®^ 100 sodium-form resin, 50–100 mesh (dry) (Sigma-Aldrich, St. Louis, MO, USA; catalog no. C7901). MICs were interpreted according to the Clinical and Laboratory Standards Institute guidelines (CLSI M100, 32st ed, 2022) (Clinical and Laboratory Standards Institute (CLSI), 2022), except for tigecycline and colistin. Tigecycline was interpreted according to the U.S. Food and Drug Administration (FDA) interpretive criteria (U.S. Food and Drug Administration, 2023), whereas colistin was interpreted according to EUCAST criteria (The European Committee on Antimicrobial Susceptibility Testing, 2026). For molecular epidemiological analyses only, reduced CFDC susceptibility was prespecified as an MIC of 4–16 mg/L. This threshold captured isolates with MICs of 4–8 mg/L, classified as resistant by EUCAST but susceptible or intermediate by CLSI, and was used only for epidemiological analysis. Escherichia coli ATCC 25922 was used as the quality-control strain. Carbapenem resistance was defined as meropenem or imipenem MIC ≥4 μg/mL, or ertapenem MIC ≥2 μg/mL (Wang Q. et al., 2024).

### 2.3 Whole-genome sequencing and bioinformatics

Genomic DNA was extracted from single colonies in exponential growth phase using the Omega Bio-Tek Bacterial DNA Kit (Doraville, GA, USA). DNA quality and concentration were assessed by 1% agarose gel electrophoresis, a NanoDrop 2000 spectrophotometer (Thermo Scientific, Waltham, MA, USA), and a Qubit 4 Fluorometer (Thermo Scientific, Waltham, MA, USA). Sequencing libraries were constructed using the NEBNext Ultra II DNA Library Prep Kit (New England Biolabs, Ipswich, MA, USA) and sequenced on the NovaSeq 6000 platform (Illumina, CA, USA) with paired-end reads and an average insert size of 350 bp. Low-quality reads were removed as previously described (Peng et al., 2018), and *de novo* assembly was performed using SPAdes v3.11 (Bankevich et al., 2012). MLST, serotype, antimicrobial resistance and virulence genes were identified using Kleborate (Lam et al., 2021). Previously reported mutations associated with CFDC resistance were manually inspected. Core-genome SNP analysis was performed using Snippy v4.6.0 (https://github.com/tseemann/snippy) with *K. pneumoniae* HS11286 (GenBank accession no. CP003200.1) as the reference genome, and recombinant regions were removed using Gubbins v2.3.4. Variant sites were extracted using SNP-sites v2.5.1, and a maximum-likelihood phylogenetic tree was reconstructed using IQ-TREE (Minh et al., 2020). Trees were visualized and annotated using tvBOT (Xie et al., 2023).

### 2.4 Statistical analysis

Statistical analyses were performed to evaluate associations between in-hospital mortality and patient- and bacterial-level characteristics. Categorical variables were compared using the chi-squared test, and continuous variables were analyzed using the Student’s t-test or Wilcoxon rank-sum test, as appropriate. For genomic analysis, isolates were grouped using the prespecified epidemiological threshold of cefiderocol MIC 4–16 mg/L versus ≤ 2 mg/L to assess borderline MIC elevation, without replacing CLSI clinical categories. Among patients with CRKP bloodstream infection, 30-day mortality was compared between MIC groups using the Fisher’s exact test, with unadjusted ORs and 95% CIs reported. Length of hospital stay was summarized as median (IQR) and compared using the Mann–Whitney U test. Exploratory gene-wise comparisons included antimicrobial resistance determinants, outer-membrane permeability alterations, virulence-associated loci, and iron-acquisition systems, using the chi-squared or Fisher’s exact test as appropriate. These analyses were hypothesis-generating and not intended to establish causality. Variables with *P* ≤ 0.05 and higher prevalence in the reduced-susceptibility group were considered for multivariable analysis. Because *iucA*, *iucB*, *iucC*, *iucD*, and *iutA* constitute the same aerobactin biosynthesis and uptake locus, they were represented by a single binary aerobactin variable. The final model included *bla*_NDM-1_, *bla*_SHV-12_, and aerobactin, yielding approximately 11.3 events per predictor. Pairwise Phi coefficients, variance inflation factors, and tolerance values were used to assess multicollinearity. Adjusted odds ratios and 95% confidence intervals were estimated using multivariable binary logistic regression.

All analyses were performed using GraphPad Prism v9.0 (GraphPad Software, La Jolla, CA, USA) or SAS version 9.4 (SAS Institute, Cary, NC, USA). A two-sided P value < 0.05 was considered statistically significant.

## 3 Results

### 3.1 Clinical relevance of CRKP bloodstream infection

During the study period, 389 episodes of *K. pneumoniae* BSI were included. CRKP accounted for 83 episodes (21.3%). Compared with CSKP bloodstream infection, CRKP bloodstream infection was associated with markedly higher 30-day mortality [31.3% (26/83) vs 2.9% (9/306), *P* < 0.001] and in-hospital mortality [41.0% (34/83) vs 8.5% (26/306), *P* < 0.001] (**Supplementary Table 1**). In the overall cohort, CRKP infection remained independently associated with in-hospital mortality after adjustment for clinical severity and infection source (**Table 1**). Among patients with CRKP BSI, respiratory-source bacteraemia (OR 5.38, 95% CI 1.70-17.05; *P* = 0.004) and chronic kidney disease (OR 3.58, 95% CI 1.04-12.30; *P* = 0.043) remained independent predictors of in-hospital death (**Supplementary Table 2**).

**Table 1:** Multivariable logistic regression analysis of risk factors associated with in-hospital mortality among patients with *K. pneumoniae* bloodstream infection.

Variables	Odds ratio	95% CI^a^	*P* value^b^
Shock	2.91	1.43–5.93	0.003
ICU admission	5.44	2.03–14.55	< 0.001
Respiratory source	7.39	2.63–20.75	< 0.001
Carbapenem resistance	4.18	1.55–11.32	0.005

^a^ CI, confidence interval. ^b^Multivariable logistic regression as indicated; P value < 0.05 was considered statistically significant.

### 3.2 *In vitro* activity of cefiderocol against CRKP bloodstream isolates

**Figure 1:**
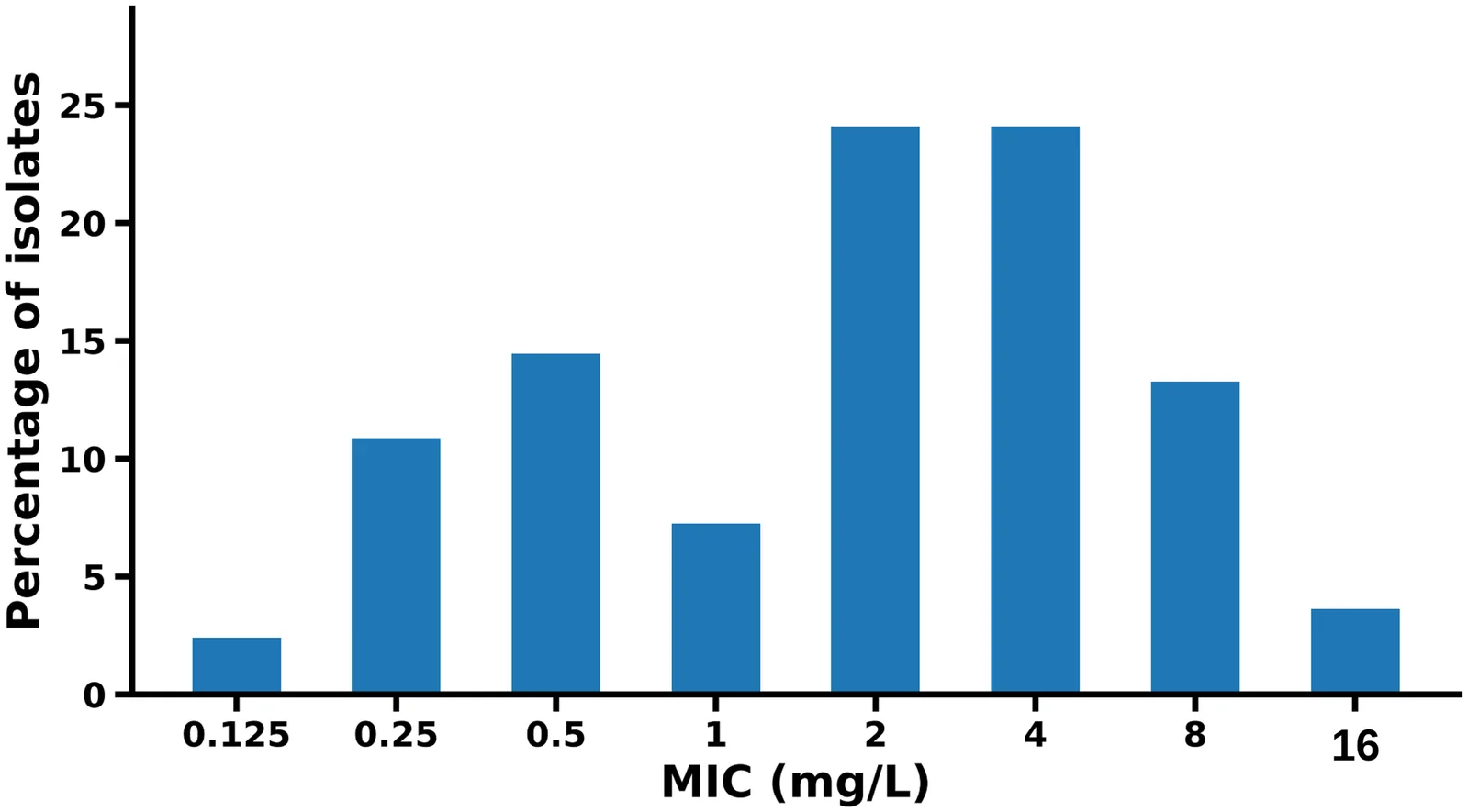
Distribution of cefiderocol minimum inhibitory concentrations among carbapenem-resistant *Klebsiella pneumoniae* (CRKP) bloodstream isolates. Bars indicate the percentage of isolates at each cefiderocol MIC. CFDC, cefiderocol; MIC, minimum inhibitory concentration.

As CFDC had not yet entered clinical use in our region, we further evaluated its *in vitro* activity against all 83 CRKP bloodstream isolates. Among the available agents, colistin showed the highest activity (susceptibility rate 94.0%, 78/83), followed by tigecycline (90.4%, 75/83). According to CLSI breakpoints for Enterobacterales, the susceptibility rate of CFDC was 83.1% (69/83), comparable to that of CZA (81.9%, 68/83) (**Supplementary Figure 1**). The MIC range of CFDC was 0.125–16 mg/L. MIC values of 2 mg/L and 4 mg/L were the most common, each accounting for 24.1% (20/83) of isolates. Of these, three were resistant isolates (all with MIC 16 mg/L), and 11 had an MIC of 8 mg/L, resulting in an overall non-susceptibility rate of 16.9% (14/83) (**Figure 1**). No high-level CFDC resistance was observed. The MIC_50_ and MIC_90_ were 4 mg/L and 8 mg/L, respectively.

### 3.3 Baseline genomic characteristics of CRKP isolates

**Figure 2:**
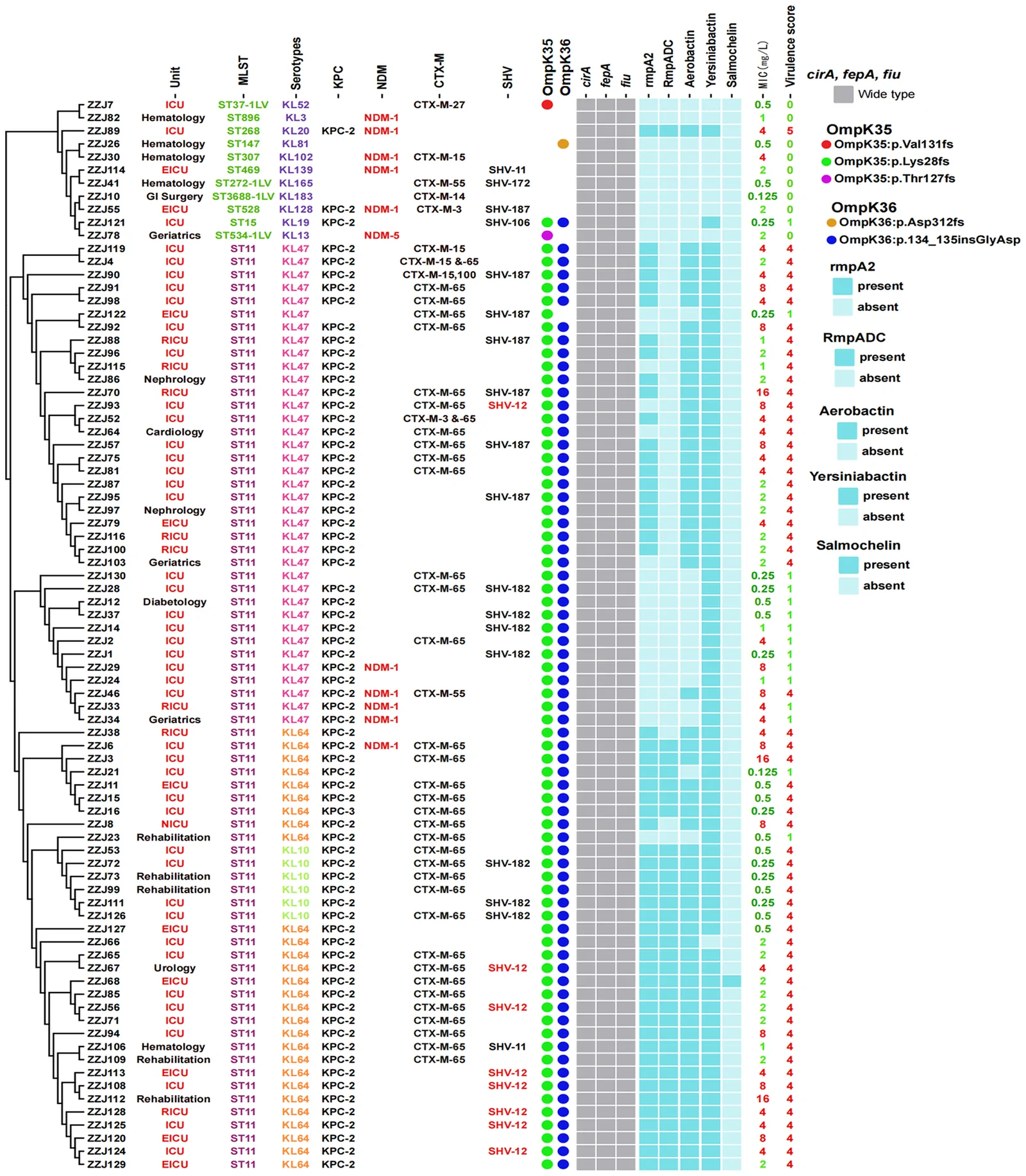
Core-genome phylogeny of 82 CRKP bloodstream isolates showing ward distribution, MLST, serotype, selected resistance and virulence determinants, porin and iron-uptake receptor alterations, cefiderocol (CFDC) MICs, and virulence scores.

Whole-genome sequencing identified 82 isolates as *K. pneumoniae* and one as *Klebsiella quasipneumoniae*. These 83 isolates were therefore considered members of the carbapenem-resistant *K. pneumoniae* species complex for genomic analyses. The genetic relatedness among the 82 K*. pneumoniae* isolates is presented in **Figure 2**. MLST identified 13 sequence types (STs), with ST11 predominating (71/83, 85.5%). The remaining isolates were distributed across 12 non-ST11 STs. Fifteen KL types were identified, among which KL47 was the most common (37/83, 44.6%), followed by KL64 (28/83, 33.7%) and KL10 (6/83, 7.2%). ST11 isolates were distributed across multiple wards, whereas non-ST11 isolates were more often confined to a single ward (**Figure 2**). Among the 83 isolates, *bla*_KPC-2_ was the predominant carbapenemase gene (65 isolates, 78.3%), followed by co-harbored *bla*_KPC-2_ and *bla*_NDM-1_ (7, 8.4%), *bla*_NDM-1_ alone (3, 3.6%), *bla*_NDM-5_ (1, 1.2%), and *bla*_NDM-1_ together with *bla*_IMP-4_ (1, 1.2%); six isolates (7.2%) were non-carbapenemase-producing (**Supplementary Figure 2**).

### 3.4 CFDC MIC distribution across capsular types and virulence backgrounds

**Figure 3:**
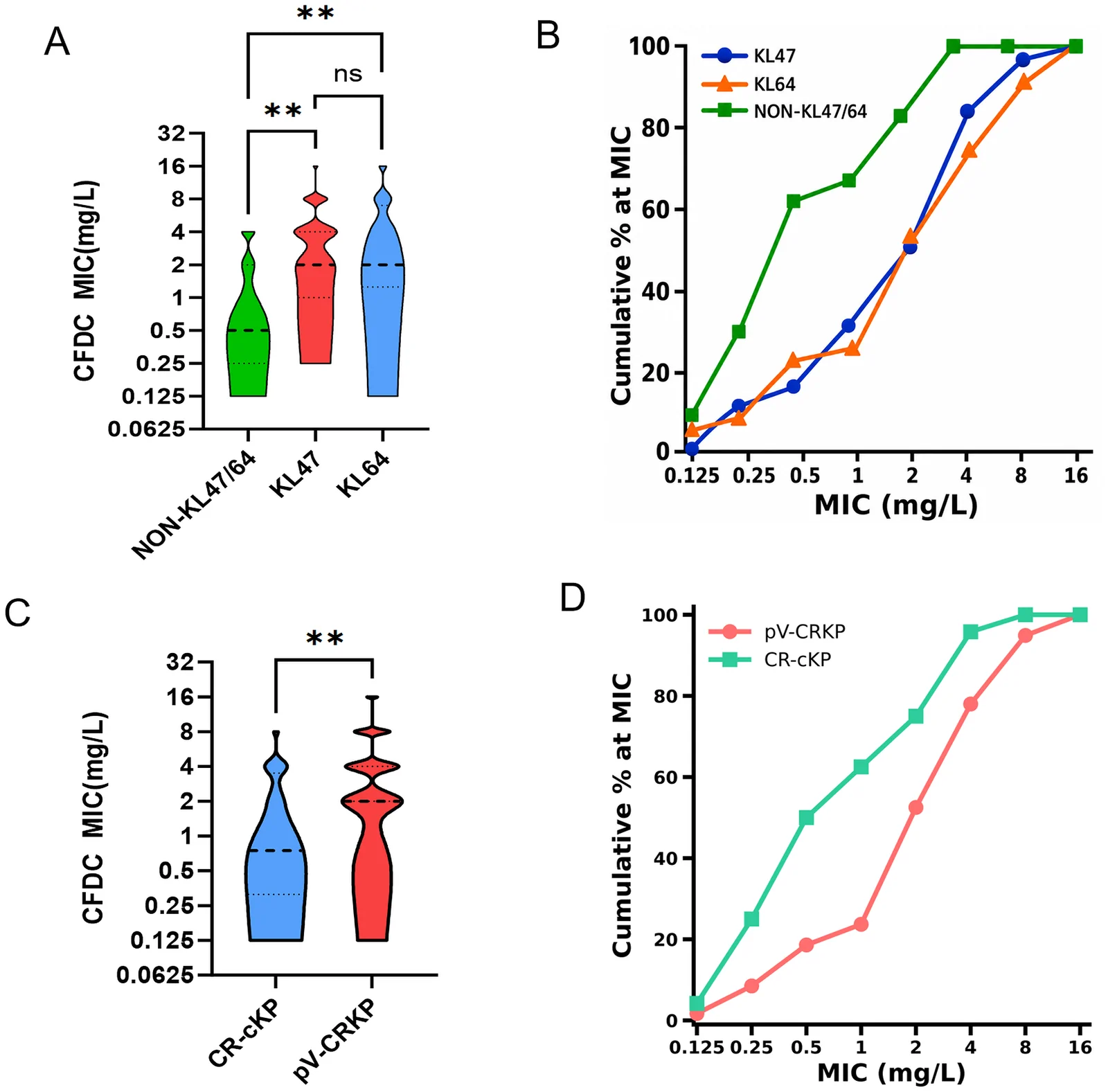
Cefiderocol MIC distributions across capsular and virulence-associated backgrounds among CRKP bloodstream isolates. **(A)** MIC distributions among non-KL47/KL64, KL47, and KL64 isolates; **(B)** corresponding cumulative inhibition curves; **(C)** MIC distributions between CR-cKP and pV-CRKP; and **(D)** corresponding cumulative inhibition curves. Horizontal lines indicate the median and interquartile range. **P < 0.01; ns, not significant. CFDC, cefiderocol; MIC, minimum inhibitory concentration; CR-cKP, classical CRKP; pV-CRKP, CRKP carrying virulence-plasmid-associated markers.

Comparison of CFDC MIC distributions across capsular types and virulence backgrounds showed that KL47 and KL64 isolates had a right-shifted CFDC MIC distribution, with median MICs of 2 mg/L for both, substantially higher than the 0.5 mg/L observed in non-KL47/KL64 isolates (both *P* < 0.01) (**Figure 3A**). Consistently, the cumulative inhibition curves for KL47 and KL64 isolates were uniformly downward-shifted compared with those for non-KL47/KL64 isolates (**Figure 3B**). According to the definition proposed by Gu et al. (2018), plasmid-virulent CRKP (pV-CRKP) was defined as *K. pneumoniae* carrying multiple virulence plasmid-associated markers, including *iucA*, *rmpA*/*rmpA2*, and/or *iroB*. Fifty-eight isolates (70.7%) were classified as pV-CRKP, whereas 24 (29.3%) were classified as classical CRKP. The median CFDC MIC of pV-CRKP was 2 mg/L, significantly higher than that of classical CRKP (0.5 mg/L; *P* < 0.01) (**Figure 3C**). The cumulative inhibition curve was also shifted downward in pV-CRKP, indicating reduced susceptibility to CFDC (**Figure 3D**).

### 3.5 Association of resistance, virulence, and iron-uptake determinants with reduced CFDC susceptibility

For molecular epidemiological analyses, however, isolates with CFDC MICs of 4–16 mg/L were prespecified as the reduced-susceptibility group. By this analytic definition, 34/83 (41.0%) isolates showed reduced CFDC susceptibility, including all 14 CLSI non-susceptible isolates and 20 isolates with an MIC of 4 mg/L that remained formally susceptible by CLSI but fell within the biologically relevant borderline interval (**Figure 1**). Using the prespecified analytic threshold, the 83 CRKP isolates were classified into a reduced-susceptibility group (MIC 4–16 mg/L, 34/83, 41.0%) and a lower-MIC comparator group (MIC ≤2 mg/L, 49/83, 59.0%). Exploratory gene-wise comparisons showed that *bla*_NDM-1_ (23.5% versus 6.1%, *P* = 0.044), *bla*_SHV-12_ (20.6% versus 2.0%, *P* = 0.007), and the aerobactin locus (82.4% versus 61.2%, *P* = 0.039) were more prevalent in the reduced-susceptibility group (**Supplementary Table 3**). The aerobactin-associated genes *iucA*, *iucB*, *iucC*, *iucD*, and *iutA* showed concordant enrichment but were treated collectively as components of the same biosynthesis and uptake locus rather than as independent predictors. Conversely, *bla*_SHV-182_ was detected only in the lower-MIC group (0% versus 14.3%, *P* = 0.038). Most other resistance determinants, virulence-associated loci, iron-acquisition systems, and porin-related alterations did not differ significantly between groups (**Supplementary Table 3**).

No evident multicollinearity was observed among *bla*_NDM-1_, *bla*_SHV-12_, and the aerobactin locus (**Supplementary Tables 4**, **S5**). In multivariable logistic regression, *bla*_NDM-1_, *bla*_SHV-12_, and aerobactin remained independently associated with reduced CFDC susceptibility (**Table 2**). However, the wide confidence intervals indicate considerable imprecision, and these findings should be interpreted as exploratory statistical associations rather than causal or mechanistic effects. Notably, the core iron-uptake receptor genes *cirA*, *fepA*, and *fiu* were wild type in all isolates, and porin-related variants were comparably distributed between groups (**Supplementary Table 3**).

**Table 2:** Multivariable logistic regression analysis of factors associated with reduced cefiderocol susceptibility.

Variables	Odds ratio	95% CI	*P* value^a^
*bla*_NDM-1_	33.5	3.19–351.28	0.003
*bla*_SHV-12_	11.14	1.27–98.09	0.03
Aerobactin	11.74	1.46–94.25	0.021

^a^ Multivariable logistic regression as indicated; P value < 0.05 was considered statistically significant.

### 3.6 Exploratory associations between cefiderocol MIC grouping and clinical outcomes

In exploratory analyses among the 83 patients with CRKP bloodstream infection, 30-day mortality was numerically higher in the CFDC MIC 4–16 mg/L group than in the MIC ≤2 mg/L group, although the difference was not statistically significant (38.2% [13/34] vs 26.5% [13/49]; OR, 1.71; 95% CI, 0.67–4.38; *P* = 0.337). The median length of hospital stay was 29.0 days (IQR, 18.2–52.0) and 41.0 days (IQR, 24.0–74.0), respectively, with no significant between-group difference (*P* = 0.173) (**Table 3**).

**Table 3:** Association between cefiderocol MIC category and clinical outcomes among 83 patients with CRKP bloodstream infection.

Clinical outcome	MIC 4–16 mg/L (n = 34)	MIC ≤2 mg/L (n = 49)	OR^a^ (95% CI)^b^	*P* value
30-day mortality, n (%)	13 (38.2)	13 (26.5)	1.71 (0.67–4.38)	0.337
Length of hospital stay, days, median (IQR)^c^	29.0 (18.2–52.0)	41.0 (24.0–74.0)	—	0.173

^a^ OR, odds ratio; ^b^CI, confidence interval; ^c^IQR, interquartile range. P value < 0.05 was considered statistically significant.

## 4 Discussion

This study characterized CFDC susceptibility, genomic features, and clinical data among CRKP bloodstream isolates collected before the local clinical introduction of CFDC. Although CFDC retained *in vitro* activity against the majority of isolates by CLSI criteria (83.1% susceptible), the MIC_50_ and MIC_90_ were 4 mg/L and 8 mg/L, respectively, and 41.0% of isolates clustered within the reduced-susceptibility range of 4–16 mg/L. These findings indicate that low-level decrements in CFDC susceptibility can emerge in the absence of prior drug selection pressure, most plausibly reflecting the intrinsic influence of dominant resistant clones and their distinctive genomic architectures (Hong et al., 2024; Yang et al., 2024).

The substantial clinical burden of CRKP bloodstream infection was further corroborated in this study (Wang M. et al., 2022; Li et al., 2023). CRKP accounted for 21.3% of all *K. pneumoniae* BSIs and remained an independent predictor of in-hospital mortality after adjustment for infection severity and source of bacteraemia. Because the analysis was restricted to bloodstream isolates from clinically significant invasive infections, it provides a focused complement to surveillance studies based on mixed infection and colonization sources.

Consistent with prior large-scale surveillance programs, CFDC maintained high *in vitro* activity against the majority of CRKP isolates in this cohort (Hackel et al., 2017; Takemura et al., 2023). However, categorical interpretation differed substantially by breakpoint system. Of the 34 isolates with MICs of 4–16 mg/L, 20 with an MIC of 4 mg/L were susceptible by CLSI but resistant by EUCAST, 11 with an MIC of 8 mg/L were intermediate by CLSI but resistant by EUCAST, and three with an MIC of 16 mg/L were resistant under both systems. Accordingly, the CLSI non-susceptibility rate was 16.9% (14/83), whereas the EUCAST resistance rate was 41.0% (34/83). This discrepancy is consistent with previous observations that cefiderocol non-susceptibility rates among NDM-producing Klebsiella bloodstream isolates vary substantially according to the interpretive system applied (Isler et al., 2023). The 41.0% reduced-susceptibility proportion should therefore be interpreted as an epidemiological indicator of a right-shifted MIC distribution rather than as a resistance rate based on a single breakpoint system. For isolates with MICs of 4–8 mg/L, the numerical MIC and interpretive standard should be reported together, as current evidence remains insufficient to determine which system better predicts clinical outcomes.

To explore the clinical relevance of CFDC MIC elevation, we compared 30-day mortality and length of hospital stay between the prespecified MIC groups. Although 30-day mortality was numerically higher and hospital stay shorter in the MIC 4–16 mg/L group, neither difference was statistically significant. Because CFDC was not used during the study period, these findings reflect baseline MIC–outcome associations rather than treatment effects. Residual confounding and the influence of early mortality on hospital stay cannot be excluded; therefore, the results should be considered hypothesis-generating and validated in larger CFDC-treated cohorts.

In this cohort, ST11 was the overwhelmingly dominant sequence type, with KL47 and KL64 as the predominant capsular loci. KL47/KL64 strains exhibited a significantly rightward-shifted CFDC MIC distribution relative to non-KL47/KL64 isolates, and pV-CRKP demonstrated consistently higher MICs than classical CRKP. ST11 constitutes the predominant epidemic lineage of CRKP in China, and ST11-KL64 together with its related sublineages has undergone marked expansion in recent years, increasingly harboring convergent combinations of resistance determinants, virulence plasmids, and enhanced nosocomial transmissibility (Wang Q. et al., 2024; Wang R. et al., 2024; Chen et al., 2025). More broadly, the international spread of KPC/NDM co-producing *K. pneumoniae* and the emergence of hv-CRKP/CR-hvKP in multiple regions indicate that resistance and hypervirulence are no longer separate evolutionary trajectories, but increasingly convergent epidemiological processes (Wu et al., 2024; Zhang et al., 2025). Consequently, these results indicate that reduced CFDC susceptibility may be enriched in specific high-risk ST11 backgrounds rather than determined by a single resistance gene alone.

In the multivariable analysis, *bla*_NDM-1_, *bla*_SHV-12_, and the aerobactin locus remained associated with reduced CFDC susceptibility. Pairwise Phi coefficients, VIFs, and tolerance values showed no evident multicollinearity among these predictors, although this does not exclude residual confounding by lineage or other co-occurring genomic features.

The relationship between NDM and elevated CFDC MICs is supported by numerous studies. Compared with other carbapenemases such as KPC, OXA-48, IMP or VIM, NDM-producing organisms generally exhibit higher CFDC MICs and higher rates of non-susceptibility (Yang et al., 2024; Lu et al., 2025). The enrichment of *bla*_NDM-1_ in the reduced-susceptibility group and its retained association in multivariable analysis suggest that *bla*_NDM-1_-positive genomic backgrounds may be associated with elevated CFDC MICs in CRKP. Accordingly, for patients with *bla*_NDM_-positive CRKP infections, clinical deployment of CFDC should be guided by reference broth microdilution susceptibility testing combined with comprehensive molecular characterization (Karakonstantis et al., 2022).

The association with *bla*_SHV-12_ is also compatible with prior functional evidence that *bla*_SHV-12_ amplification or increased expression can promote heteroresistance or higher cefiderocol MICs, particularly when combined with other resistance or permeability mechanisms (Wang Q. et al., 2022; Liu et al., 2024; Lan et al., 2025). However, copy number, transcription, and enzyme abundance were not measured in the present study. The finding therefore supports prioritization of SHV-12-positive isolates for further investigation but does not demonstrate that SHV-12 expression caused the observed MIC shift.

Aerobactin represents a cornerstone iron-acquisition determinant of hypervirulent *K. pneumoniae* and is characteristically encoded on virulence plasmids that define the pV-CRKP phenotype (Russo et al., 2021; Zhao et al., 2023). A recent Chinese study demonstrated that CFDC exhibited diminished *in vitro* activity against CR-hvKp compared with classical CRKP, an effect attributed at least in part to the markedly elevated siderophore output of hypervirulent strains (Zhao et al., 2023). CFDC relies on bacterial iron-uptake systems for periplasmic entry, yet the impact of different siderophores and their receptors on CFDC uptake is inconsistent. Recent work has also shown that plasmid-encoded iron-uptake modules can influence CFDC MICs in KPC-producing *K. pneumoniae* (Polani et al., 2025). In the present study, aerobactin carriage was significantly enriched among bloodstream CRKP isolates with reduced CFDC susceptibility. Whether this association reflects a direct mechanistic effect of aerobactin-mediated competitive siderophore interference or merely serves as a genomic marker of the high-risk ST11 virulence-plasmid background remains an open question warranting further investigation. Accordingly, aerobactin should be interpreted here as an exploratory genomic marker. Direct mechanistic assessment will require long-read plasmid reconstruction, transcriptional and siderophore-production analyses, iron-uptake phenotyping, and experiments using isogenic strains.

Of note, no inactivating mutations were identified in the canonical catecholate siderophore receptors *cirA*, *fepA*, or *fiu*, and porin-associated variants did not differ significantly between susceptibility groups. CirA deficiency is an established determinant of impaired CFDC periplasmic uptake and, when superimposed upon NDM production or high-level β-lactamase expression, can precipitate overt CFDC resistance or heteroresistance (Lan et al., 2022; Hong et al., 2024). The predominance of low-level MIC right-shifts rather than frank high-level resistance in the absence of classical receptor-inactivating mutations implies that the susceptibility decrements observed in this cohort are governed by a constellation of interacting factors: the β-lactamase repertoire, the ST11 clonal scaffold, and virulence-plasmid-encoded iron-acquisition modules operating in concert, rather than by loss of any single siderophore transporter.

These findings further underscore the mechanistic distinction between high-level CFDC resistance and low-level MIC elevation. High-level resistance typically involves the superimposition of multiple factors, including NDM, CirA truncation, TonB-dependent transport impairment, porin alterations, and high-level β-lactamase expression (Karakonstantis et al., 2022). Such borderline phenotypes may depend on the cumulative effect of several weak contributors, including NDM, SHV-12, the KPC background, virulence-plasmid-associated iron-uptake systems, and the clonal characteristics of ST11-KL47/KL64. From a clinical perspective, these isolates risk being miscategorized as fully susceptible under routine breakpoint interpretation, yet they may represent an evolutionarily poised substrate from which frank resistance could emerge upon CFDC selective pressure.

This study also carries implications for clinical susceptibility testing. Standard CFDC testing relies on iron-depleted cation-adjusted Mueller–Hinton broth, and results are susceptible to methodological and iron-content variations (Bonnin et al., 2022). For isolates with MICs of 4–8 mg/L, the specific numerical value should be noted rather than relying solely on categorical susceptible or resistant designations. For ST11-KL47/KL64, pV-CRKP, *bla*_NDM_-positive, or *bla*_SHV-12_-positive isolates, reference broth microdilution should be used for verification when necessary, and results should be interpreted in conjunction with carbapenemase type and key genetic background (Irigoyen-von-Sierakowski et al., 2024).

This study has several limitations. First, its single-center design and the predominance of ST11 may limit the generalizability of the findings and may not fully exclude residual confounding related to bacterial lineage. Second, the reduced-susceptibility group was defined for epidemiological analysis and included isolates across different clinical breakpoint categories; it was not intended to redefine clinical susceptibility. Third, the gene-by-gene comparisons were exploratory and hypothesis-generating and should not be interpreted as evidence of causal mechanisms. Fourth, short-read sequencing could not fully resolve the structures of resistance and virulence plasmids. In addition, transcriptional analyses, siderophore or iron-uptake assays, and functional experiments in isogenic backgrounds were not performed. These limitations are compatible with the study’s intended role as a pre-introduction surveillance baseline.

## 5 Conclusion

In conclusion, borderline CFDC MIC elevation was already present among CRKP bloodstream isolates before local clinical introduction of the drug and was concentrated within ST11-KL47/KL64 and virulence-plasmid-associated backgrounds. *bla*_NDM-1_, *bla*_SHV-12_, and the aerobactin locus were statistically associated with this phenotype, but these associations do not establish causality. The study provides a pre-introduction, bloodstream-specific surveillance baseline and supports targeted monitoring of high-risk CRKP populations together with careful reporting of numerical CFDC MICs.

## Data Availability

The data presented in the study are deposited in the NCBI repository under BioProject accession number PRJNA1474708 and GenBank accession numbers JBYVGP000000000–JBYVJT000000000.
