# Supplementary material for: Genetic characterization of carbapenem-resistant Klebsiella pneumoniae bloodstream isolates with reduced susceptibility to cefiderocol
Source: Front Cell Infect Microbiol. 2026 Aug 27;16:1890499. doi: 10.3389/fcimb.2026.1890499 (PMC13557972; doi:10.3389/fcimb.2026.1890499)
Supplement: Supplementary file 1 [file Table1.docx]

**Supplementary Figures and Tables.**

**Figure S1. Antimicrobial susceptibility profile of carbapenem-resistant *K. pneumoniae* species complex bloodstream isolates.**

Stacked bar chart showing the proportions of susceptible (S), intermediate/susceptible-dose dependent (I/SDD), and resistant (R) isolates for each antimicrobial agent. TZP, piperacillin/tazobactam; CRO, ceftriaxone; CAZ, ceftazidime; FEP, cefepime; SCF, cefoperazone/sulbactam; ATM, aztreonam; IPM, imipenem; MEM, meropenem; ETP, ertapenem; LEV, levofloxacin; CIP, ciprofloxacin; AK, amikacin; TOB, tobramycin; GEN, gentamicin; SXT, trimethoprim/sulfamethoxazole; TGC, tigecycline; COL, colistin; CZA, ceftazidime/avibactam; FDC, cefiderocol.

**Figure S2. Distribution of carbapenemase genes among 83 carbapenem-resistant *K. pneumoniae* species complex bloodstream isolates.**

**Table S1. Baseline Characteristics of Patients with CSKP and CRKP-BSI.**

| **Variables** | **Overall (N=389)** | **CRKP (N=83)** | **CSKP (N=306)** | ***P* value^a^** |
| --- | --- | --- | --- | --- |
| Age, years (IQR) | 67.5 (55.3,78.8) | 74 (65–83) | 66 (52–75) | <0.001 |
| Sex (Female) | 121 (31.1%) | 15 (18.1%) | 106 (34.6%) | 0.006 |
| Charlson Comorbidity (CCI) >3 | 88 (22.6%) | 35 (42.2%) | 53 (17.3%) | <0.001 |
| Chronic liver disease | 52 (13.4%) | 8 (9.6%) | 44 (14.4%) | 0.345 |
| Chronic renal disease | 33 (8.5%) | 18 (21.7%) | 15 (4.9%) | <0.001 |
| Cancer | 103 (26.5%) | 26 (31.3%) | 77 (25.2%) | 0.323 |
| Solid tumor | 72 (18.5%) | 19 (22.9%) | 53 (17.3%) | 0.317 |
| Hematologic malignancy | 33 (8.5%) | 7 (8.4%) | 26 (8.5%) | 1.000 |
| Diabetes | 177 (45.5%) | 40 (48.2%) | 137 (44.8%) | 0.667 |
| Respiratory disease | 134 (34.4%) | 51 (61.4%) | 83 (27.1%) | <0.001 |
| Cardiovascular disease | 140 (36.0%) | 45 (54.2%) | 95 (31.0%) | <0.001 |
| Neurological disease | 133 (34.2%) | 43 (51.8%) | 90 (29.4%) | <0.001 |
| Immunosuppressed | 82 (21.1%) | 19 (22.9%) | 63 (20.6%) | 0.761 |
| Shock | 89 (22.9%) | 31 (37.3%) | 58 (19.0%) | <0.001 |
| Hospital acquired | 184 (47.3%) | 75 (90.4%) | 109 (35.6%) | <0.001 |
| ICU^a^ admission | 201 (51.7%) | 63 (75.9%) | 138 (45.1%) | <0.001 |
| Infection source: Abdominal | 29 (7.5%) | 14 (16.9%) | 15 (4.9%) | <0.001 |
| Infection source: Respiratory | 57 (14.7%) | 24 (28.9%) | 33 (10.8%) | <0.001 |
| Infection source: Urinary | 65 (16.7%) | 13 (15.7%) | 52 (17.0%) | 0.903 |
| Infection source: Central line | 45 (11.6%) | 22 (26.5%) | 23 (7.5%) | <0.001 |
| Infection source: Unidentified | 193 (49.6%) | 10 (12.0%) | 183 (59.8%) | <0.001 |
| Incorrect first Abx | 64 (16.5%) | 38 (45.8%) | 26 (8.5%) | <0.001 |
| 30 days death | 35 (9.0%) | 26 (31.3%) | 9 (2.9%) | <0.001 |
| Hospital death | 60 (15.4%) | 34 (41.0%) | 26 (8.5%) | <0.001 |

CSKP, Carbapenem-susceptible *K. pneumoniae*; CRKP, carbapenem-resistant *K.pneumoniae*; BSI, bloodstream infection. ^a^χ^2^ or Fisher exact test was used for categorical variables, and Kruskal-Wallis test was used for continuous variables; P value < 0.05 was considered statistically significant.

**Table S2. Multivariable logistic regression analysis of risk factors associated with in-hospital mortality among patients with CRKP bloodstream infection.**

| **Variables** | **Odds ratio** | **95% CI^a^** | ***P* value^b^** |
| --- | --- | --- | --- |
| Respiratory source | 5.38 | 1.70–17.05 | 0.004 |
| chronic kidney disease | 3.58 | 1.04–12.30 | 0.043 |

^a^CI, confidence interval. ^b^Multivariate logistic regression as indicated; P value < 0.05 was considered

statistically significant.

**Table S3. Association of resistance, virulence, and iron-uptake determinants with reduced cefiderocol susceptibility of CRKP bloodstream isolates.**

| **Gene** | **CFDC^a^ >= 4 mg/L (n = 34)** | **CFDC <= 2 mg/L (n = 49)** | ***P* value^b^** |
| --- | --- | --- | --- |
| *ARR-3* | 2 (5.9%) | 6 (12.2%) | 0.462 |
| *aac(3)-IIa* | 1 (2.9%) | 0 (0.0%) | 0.410 |
| *aac(3)-IId* | 1 (2.9%) | 4 (8.2%) | 0.644 |
| *aac(6')-Ib-cr* | 2 (5.9%) | 2 (4.1%) | 1.000 |
| *aadA16* | 0 (0.0%) | 1 (2.0%) | 1.000 |
| *aadA22* | 0 (0.0%) | 1 (2.0%) | 1.000 |
| *aadA2* | 6 (17.6%) | 10 (20.4%) | 0.754 |
| *ant(3'')-Ia* | 0 (0.0%) | 0 (0.0%) | 1.000 |
| *aph(3'')-Ib* | 3 (8.8%) | 5 (10.2%) | 1.000 |
| *aph(3')-Ia* | 4 (11.8%) | 7 (14.3%) | 1.000 |
| *aph(6)-Id* | 3 (8.8%) | 6 (12.2%) | 0.731 |
| *armA* | 6 (17.6%) | 9 (18.4%) | 0.933 |
| *bla*_CMY-6_ | 1 (2.9%) | 0 (0.0%) | 0.410 |
| *bla*_CTX-M-105_ | 0 (0.0%) | 1 (2.0%) | 1.000 |
| *bla*_CTX-M-14_ | 0 (0.0%) | 1 (2.0%) | 1.000 |
| *bla*_CTX-M-15_ | 4 (11.8%) | 1 (2.0%) | 0.154 |
| *bla*_CTX-M-27_ | 0 (0.0%) | 1 (2.0%) | 1.000 |
| *bla*_CTX-M-3_ | 1 (2.9%) | 1 (2.0%) | 1.000 |
| *bla*_CTX-M-55_ | 1 (2.9%) | 1 (2.0%) | 1.000 |
| *bla*_CTX-M-65_ | 17 (50.0%) | 20 (40.8%) | 0.408 |
| *bla*_DHA-1_ | 6 (17.6%) | 11 (22.4%) | 0.594 |
| *bla*_IMP-4_ | 1 (2.9%) | 0 (0.0%) | 0.410 |
| *bla*_KPC-2_ | 32 (94.1%) | 39 (79.6%) | 0.110 |
| *bla*_KPC-3_ | 0 (0.0%) | 1 (2.0%) | 1.000 |
| ***bla***_NDM-1_ | 8 (23.5%) | 3 (6.1%) | **0.044** |
| *bla*_NDM-5_ | 0 (0.0%) | 1 (2.0%) | 1.000 |
| *bla*_OXA-10_ | 5 (14.7%) | 11 (22.4%) | 0.379 |
| *bla*_OXA-1_ | 2 (5.9%) | 1 (2.0%) | 0.565 |
| *bla*_SHV-106_ | 0 (0.0%) | 1 (2.0%) | 1.000 |
| *bla*_SHV-11_ | 0 (0.0%) | 2 (4.1%) | 0.510 |
| ***bla***_SHV-12_ | 7 (20.6%) | 1 (2.0%) | **0.007** |
| *bla*_SHV-172_ | 0 (0.0%) | 1 (2.0%) | 1.000 |
| ***bla***_SHV-182_ | 0 (0.0%) | 7 (14.3%) | **0.038** |
| *bla*_SHV-187_ | 0 (0.0%) | 4 (8.2%) | 0.141 |
| *bla*_TEM-1B_ | 6 (17.6%) | 8 (16.3%) | 0.874 |
| *bla*_VEB-3_ | 1 (2.9%) | 0 (0.0%) | 0.410 |
| *catA2* | 3 (8.8%) | 4 (8.2%) | 1.000 |
| *catB8* | 1 (2.9%) | 1 (2.0%) | 1.000 |
| *cmlA1* | 5 (14.7%) | 12 (24.5%) | 0.277 |
| *dfrA12* | 6 (17.6%) | 6 (12.2%) | 0.537 |
| *dfrA14* | 7 (20.6%) | 15 (30.6%) | 0.309 |
| *dfrA27* | 1 (2.9%) | 2 (4.1%) | 1.000 |
| *floR* | 6 (17.6%) | 12 (24.5%) | 0.457 |
| *fosA* | 16 (47.1%) | 25 (51.0%) | 0.723 |
| *lnu(F)* | 0 (0.0%) | 1 (2.0%) | 1.000 |
| *mcr-8* | 0 (0.0%) | 1 (2.0%) | 1.000 |
| *mph(A)* | 5 (14.7%) | 9 (18.4%) | 0.661 |
| *mph(E)* | 3 (8.8%) | 2 (4.1%) | 0.396 |
| *msr(E)* | 3 (8.8%) | 2 (4.1%) | 0.396 |
| *oqxA* | 16 (47.1%) | 21 (42.9%) | 0.705 |
| *oqxB* | 16 (47.1%) | 21 (42.9%) | 0.705 |
| *qnrB1* | 1 (2.9%) | 1 (2.0%) | 1.000 |
| *qnrB4* | 5 (14.7%) | 12 (24.5%) | 0.277 |
| *qnrB52* | 0 (0.0%) | 1 (2.0%) | 1.000 |
| *qnrS1* | 18 (52.9%) | 32 (65.3%) | 0.258 |
| *rmtB* | 17 (50.0%) | 31 (63.3%) | 0.229 |
| *rmtC* | 1 (2.9%) | 0 (0.0%) | 0.410 |
| *sul1* | 6 (17.6%) | 8 (16.3%) | 0.874 |
| *sul2* | 9 (26.5%) | 11 (22.4%) | 0.674 |
| *sul3* | 0 (0.0%) | 3 (6.1%) | 0.266 |
| *tet(A)* | 10 (29.4%) | 14 (28.6%) | 0.934 |
| *tet(D)* | 0 (0.0%) | 1 (2.0%) | 1.000 |
| *GyrA* | 32 (94.1%) | 44 (89.8%) | 0.695 |
| Omp-mutation | 32 (94.1%) | 44 (89.8%) | 0.695 |
| *rmpA2* | 24 (70.6%) | 29 (59.2%) | 0.288 |
| *rmpADC* | 12 (35.3%) | 20 (40.8%) | 0.611 |
| *entA* | 33 (97.1%) | 49 (100.0%) | 0.410 |
| *entB* | 34 (100.0%) | 49 (100.0%) | 1.000 |
| *fepC* | 34 (100.0%) | 49 (100.0%) | 1.000 |
| *fepG* | 2 (5.9%) | 2 (4.1%) | 1.000 |
| **Aerobactin** | 28 (82.4%) | 30 (61.2%) | **0.039** |
| *iucA* | 28 (82.4%) | 30 (61.2%) | 0.039 |
| *iucB* | 28 (82.4%) | 29 (59.2%) | 0.025 |
| *iucC* | 26 (76.5%) | 25 (51.0%) | 0.019 |
| *iucD* | 28 (82.4%) | 30 (61.2%) | 0.039 |
| *iutA* | 28 (82.4%) | 30 (61.2%) | 0.039 |
| ***Salmochelin*** | **1 (2.9%)** | **0 (0.0%)** | **0.410** |
| *iroB* | 1 (2.9%) | 0 (0.0%) | 0.410 |
| *iroC* | 1 (2.9%) | 0 (0.0%) | 0.410 |
| *iroD* | 1 (2.9%) | 0 (0.0%) | 0.410 |
| *iroN* | 1 (2.9%) | 0 (0.0%) | 0.410 |
| ***Yersiniabactin*** | **32 (94.1%)** | **41 (83.7%)** | **0.187** |
| *ybtA* | 32 (94.1%) | 41 (83.7%) | 0.187 |
| *ybtE* | 32 (94.1%) | 41 (83.7%) | 0.187 |
| *ybtP* | 32 (94.1%) | 41 (83.7%) | 0.187 |
| *ybtQ* | 32 (94.1%) | 41 (83.7%) | 0.187 |
| *ybtS* | 32 (94.1%) | 41 (83.7%) | 0.187 |
| *ybtT* | 32 (94.1%) | 41 (83.7%) | 0.187 |
| *ybtU* | 32 (94.1%) | 41 (83.7%) | 0.187 |
| *ybtX* | 32 (94.1%) | 41 (83.7%) | 0.187 |
| *irp1* | 32 (94.1%) | 41 (83.7%) | 0.187 |
| *irp2* | 32 (94.1%) | 41 (83.7%) | 0.187 |
| *fyuA* | 32 (94.1%) | 41 (83.7%) | 0.187 |

**^a^**CFDC, cefiderocol; ^b^P values were derived using chi-square tests or Fisher exact tests.

**Table S4. Pairwise Phi correlations among candidate predictors**

| **Variable 1** | **Variable 2** | **Phi coefficient** | **P value** |
| --- | --- | --- | --- |
| *bla*_NDM-1_ | *bla*_SHV-12_ | -0.128 | 0.245 |
| *bla*_NDM-1_ | Aerobactin locus | -0.363 | < 0.001 |
| *bla*_SHV-12_ | Aerobactin locus | 0.214 | 0.051 |

Note. Phi coefficients were used to assess pairwise associations between binary predictors. The maximum absolute Phi coefficient was 0.363. *bla*_NDM-1_ and *bla*_SHV-12_ denote the corresponding β-lactamase genes.

**Table S5. Variance inflation factor diagnostics**

| **Variable** | **VIF** | **Tolerance** | **Auxiliary-model R²** |
| --- | --- | --- | --- |
| *bla*_NDM-1_ | 1.155 | 0.866 | 0.134 |
| *bla*_SHV-12_ | 1.051 | 0.951 | 0.049 |
| Aerobactin locus | 1.191 | 0.840 | 0.160 |

Note. All VIF values were below 5 and all tolerance values were above 0.20, indicating no evident multicollinearity requiring variable exclusion.
